# MicroRNA expressions associated with progression of prostate cancer cells to antiandrogen therapy resistance

**DOI:** 10.1186/1476-4598-13-1

**Published:** 2014-01-03

**Authors:** Richard Ottman, Camha Nguyen, Robert Lorch, Ratna Chakrabarti

**Affiliations:** 1 Burnett School of Biomedical Sciences, University of Central Florida, 12722 Research Parkway, Orlando, Florida, USA

**Keywords:** MicroRNA, Prostate cancer, Antiandrogen, Androgen deprivation, Drug resistance, Expression profiling

## Abstract

**Background:**

Development of resistance to androgen deprivation therapy (ADT) is a major obstacle for the management of advanced prostate cancer. Therapies with androgen receptor (AR) antagonists and androgen withdrawal initially regress tumors but development of compensatory mechanisms including AR bypass signaling leads to re-growth of tumors. MicroRNAs (miRNAs) are small regulatory RNAs that are involved in maintenance of cell homeostasis but are often altered in tumor cells.

**Results:**

In this study, we determined the association of genome wide miRNA expression (1113 unique miRNAs) with development of resistance to ADT. We used androgen sensitive prostate cancer cells that progressed to ADT and AR antagonist Casodex (CDX) resistance upon androgen withdrawal and treatment with CDX. Validation of expression of a subset of 100 miRNAs led to identification of 43 miRNAs that are significantly altered during progression of cells to treatment resistance. We also show a correlation of altered expression of 10 proteins targeted by some of these miRNAs in these cells.

**Conclusions:**

We conclude that dynamic alterations in miRNA expression occur early on during androgen deprivation therapy, and androgen receptor blockade. The cumulative effect of these altered miRNA expression profiles is the temporal modulation of multiple signaling pathways promoting survival and acquisition of resistance. These early events are driving the transition to castration resistance and cannot be studied in already developed CRPC cell lines or tissues. Furthermore our results can be used a prognostic marker of cancers with a potential to be resistant to ADT.

## Background

Prostate cancer is the most commonly diagnosed cancer and a leading cause of cancer related death in men in developed countries. Because androgen is required for normal growth and functioning of the prostate gland and also for development of cancer androgen deprivation therapy (ADT) has become the mainstay for advanced prostate cancer [1]. Although most patients initially respond to ADT by showing low PSA values, they eventually develop more aggressive castration resistant prostate cancer (CRPC). Androgen, working through androgen receptor (AR) triggers transcriptional activation of a variety of genes that are essential for growth and survival of prostate epithelial cells. However, prolonged androgen blockade using steroidal or non-steroidal inhibitors such as, cyproterone acetate, Casodex or hydroxyl flutamide, leads to activation of various adaptive mechanisms after initial retardation of cell proliferation [2,3].

Development of resistance to ADT, which includes reduction in androgen synthesis and direct antagonism of the androgen receptors (AR), can occur as a result of high expression and activation of AR [4]. Activation of AR without androgen is through switching of AR to alternative mechanism of activation. Commonly noted mechanisms include AR gene amplification, increased coactivator expression, selection of AR gene mutation and sensitivity to growth factors and cytokines [5]. AR expression and activity also increased after long-term androgen ablation to a level that was comparable to that in parental cell lines or androgen dependent (AD) tumors prior to castration [6]. It has been proposed that the increased activity of AR in androgen independent (AI) cells or in relapsed tumors in castrated xenograft mice is mediated through ligand independent mechanism [7] or through promiscuous sensitivity of AR to other steroid hormone, growth factors or cytokines [8]. Androgen blockade therapy can accumulate mutations causing AR to become sensitive to androgen antagonists, which then act as agonists [2,9]. It is now accepted that CRPC maintains functional AR signaling [10,11] but androgen refractoriness is through an AR bypass or adaptive mechanism, which is possibly, mediated through cytokines or other survival factors. Irrespective of the specific phenotypes acquired by the AD prostate cancers, the outcome is altered expression of protein-coding genes as a whole that are responsible for progression and metastasis of prostate cancer. In addition, nonsteroidal agents such as flutamide can alter gene expression in AR negative prostate tumors [12]. Gene expression profiling in androgen dependent and androgen independent prostate cancers revealed an increasingly complex profile of gene expression in prostate cancer with respect to the status of androgen sensitivity or refractoriness [13]. However, the exact mechanism of altered gene expression in CRPC is not clear.

The role of small noncoding microRNAs (miRNAs) in regulation of gene expression, which is mediated by inhibition of translation or degradation of target mRNAs is an established phenomenon [14]. MiRNAs belong to a class of 17–22 nucleotides, which contains a specific sequence at the 5’ end and regulates translation through binding to 3’UTR of the mRNAs [14]. To date, there are 1921 distinct human miRNAs have been identified, each of which regulate multiple target mRNAs (http://www.mirbase.org Nov 2011). Genes encoding miRNAs are located in the intergenic regions or within the protein-coding genes either alone or in clusters [15]. There is now abundant evidence that aberrant expression of miRNAs occurs in diverse types of cancer including prostate cancer and during different stages of disease progression [16,17]. The role of miRNAs in regulation of post-transcriptional gene expression has been implicated in 30% of the protein-coding genes [18]. Because miRNAs can be overexpressed or down regulated in cancer cells these noncoding RNAs are designated as oncogenic miRNAs or suppressor miRNAs. Functionally, miRNAs reduce the levels of many of their target mRNAs and the amount of proteins encoded by these mRNAs [19]. Because a given miRNA may have many mRNA targets the biological effects of changes in miRNA expression is likely to be dependent on the cellular environment.

A number of studies indicated aberrant expression of miRNAs in CRPC compared to AD prostate cancer cells [20,21]. Several miRNAs, such as miR-21, miR-125 and miR-32 are directly regulated by androgens in cells and xenograft models [20,22,23]. Studies include comparative analysis between androgen sensitive (AS) and -resistant prostate cancer cells, with or without treatment of AS cells with androgens or normal vs. hormone refractory prostate cancer tissues, which only provides steady state status of miRNA and gene expression. However none of these studies provide information on the mechanism of transition of androgen-sensitive or dependent prostate cancer cells to antiandrogen resistant cells. In this study, we show, for the first time alteration in expression of miRNAs and their target proteins as the cells progress to antiandrogen resistance, some of which are not detectable in the established AI cell line.

## Results

### MicroRNA expression profile differentiates between untreated LNCaP cells and cells treated with Casodex or subjected to androgen withdrawal

**Figure 1:**
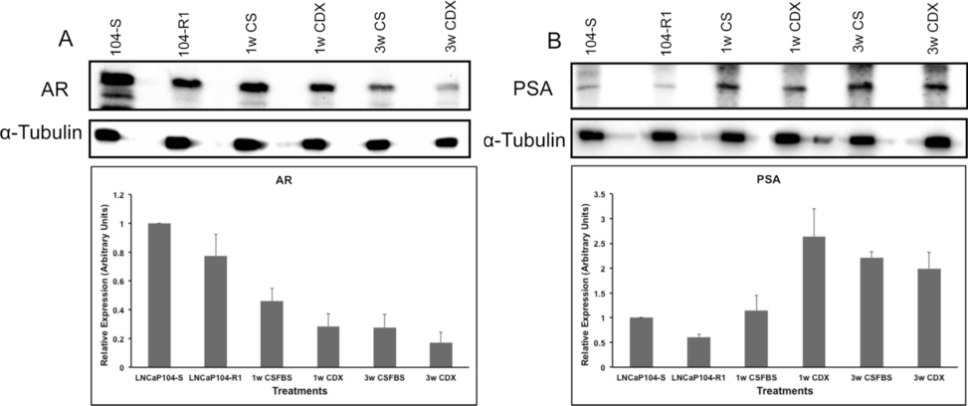
**Expression of AR and PSA in LNCaP cells.** Western blot analysis of AR **(A)** and PSA **(B)** in LNCaP cells treated with CS-FBS and CDX for different times. Upper panels: Total cell extracts were used for analysis of AR and PSA expression using anti-AR and -PSA antibodies. Lower panels: Densitometric analysis of relative expression of AR and PSA in LNCaP cells. Data represents mean ± SD of three independent experiments.

We used genome-wide miRNA array (1113 unique primers) profiling approach to identify specific miRNAs that are involved in development of resistance to Casodex (CDX). A clonal subline of LNCaP cells LNCaP-104S (-104S) and its androgen-independent derivative LNCaP-104R1 (-104R1) were used for monitoring differential expression of miRNAs upon treatment with CDX. LNCaP-104S cells are CDX-sensitive, whereas LNCaP-104R1 cells are not despite expressing AR at a basal level higher than LNCaP-104S cells [24]. LNCaP-104S cells require DHT for maintaining their AD status but when treated with CDX for 3 weeks in CS-FBS (charcoal-stripped FBS), CDX insensitive colonies develop that are independent of androgen (CDXR). During the first week of both CS-FBS and CDX treatments, LNCaP-104S cells grew without significant cell death, however, during the second week of CDX treatment about 60% cells died and detached. From the residual 40% cells, half of the cell population remained viable during the third week of CDX treatment. CS-FBS -treated cells did not show as much cell death during the second week of treatment and started to regrow during the third week of treatment. Cell morphology also changed as they are treated with CDX and CS-FBS (Additional file 1: Figure S1). Cells viable after third week of treatments were used for miRNA profiling experiments. Expression of AR in these cells showed gradual reduction as the treatment progresses, but the PSA expression increased, which suggests increased transcriptional activity of the residual AR in the treated cells (Figure 1).

We compared miRNA expressions in RNA extracts from untreated and CDX treated -104S cells at 0 hr, 1wk and 3wks and untreated -104R1 cells (Table 1). Hierarchical clustering of the normalized and log_2_ transformed expression data (Additional file 2: Table S1) showed 5 distinct clusters of miRNAs (Additional file 3: Figure S2). Comparative analysis between samples from different conditions using two samples Welch t-test with Log_10_ transformed data and *p* values of 0.05 showed significant miRNAs that are differentially expressed between conditions (Additional file 4: Table S2 and Additional file 5: Figure S3). Volcano plot (V plots) of the t-test between LNCaP-104S cells and all other samples showed 38 significant miRNAs, of which 27 miRNAs were up regulated and 11 down regulated compared to -104S (Additional file 5: Figure S3A). Comparison between untreated -104S and -104R1 cells showed 24 significant miRNAs, which includes 16 down regulated and 8 up regulated miRNAs in -104R1 (Additional file 5: Figure S3B). Differential expression of 17 significant miRNAs was observed between untreated LNCaP-104S cells and -104S cells treated with CDX, of which 13 were up regulated and 4 were down regulated CDX treated cells (Additional file 5: Figure S3C). LNCaP-104S and -104S cells treated with CSFBS also showed 9 up regulated and 5 down regulated microRNAs in CSFBS treated cells (Additional file 5: Figure S3D). Although -104R1 cells are CDX resistant there are differences in miRNA expression when -104S cells were treated with CDX (Additional file 5: Figure S3E). T-test analysis showed 24 significant miRNAs of which 18 miRNAs were up regulated and 6 down regulated in -104R1 cells. Difference in miRNA expressions was also noted between -104S cells maintained in androgen-depleted condition and AI -104R1 cells. Twenty-four significant miRNAs were identified of which 12 were up regulated and 12 down regulated in -104R1 cells (Additional file 5: Figure S3F). Comparison between androgen depletion and CDX treatment showed 5 significant miRNAs, 4 of which were up regulated and one down regulated in CDX treated cells (Additional file 5: Figure S3G).

**Table 1:** Cell lines and treatments

		**Samples**		
		**Cell line**	**Treatment**	**Time point**
Reference	0 hr	LNCaP-104S	FBS-DHT 1nM	0 hr
	1wk CSFBS	LNCaP-104S	CSFBS	1 wk
	3wks CSFBS	LNCaP-104S	CSFBS	3 wks
	1wk CDX	LNCaP-104S	CSFBS/5 μM CDX	1 wk
	3wks CDX	LNCaP-104S	CSFBS/5 μM CDX	3 wks
Test/Reference	0 hr	LNCaP-104R1	CSFBS	0 wks

**Figure 2:**
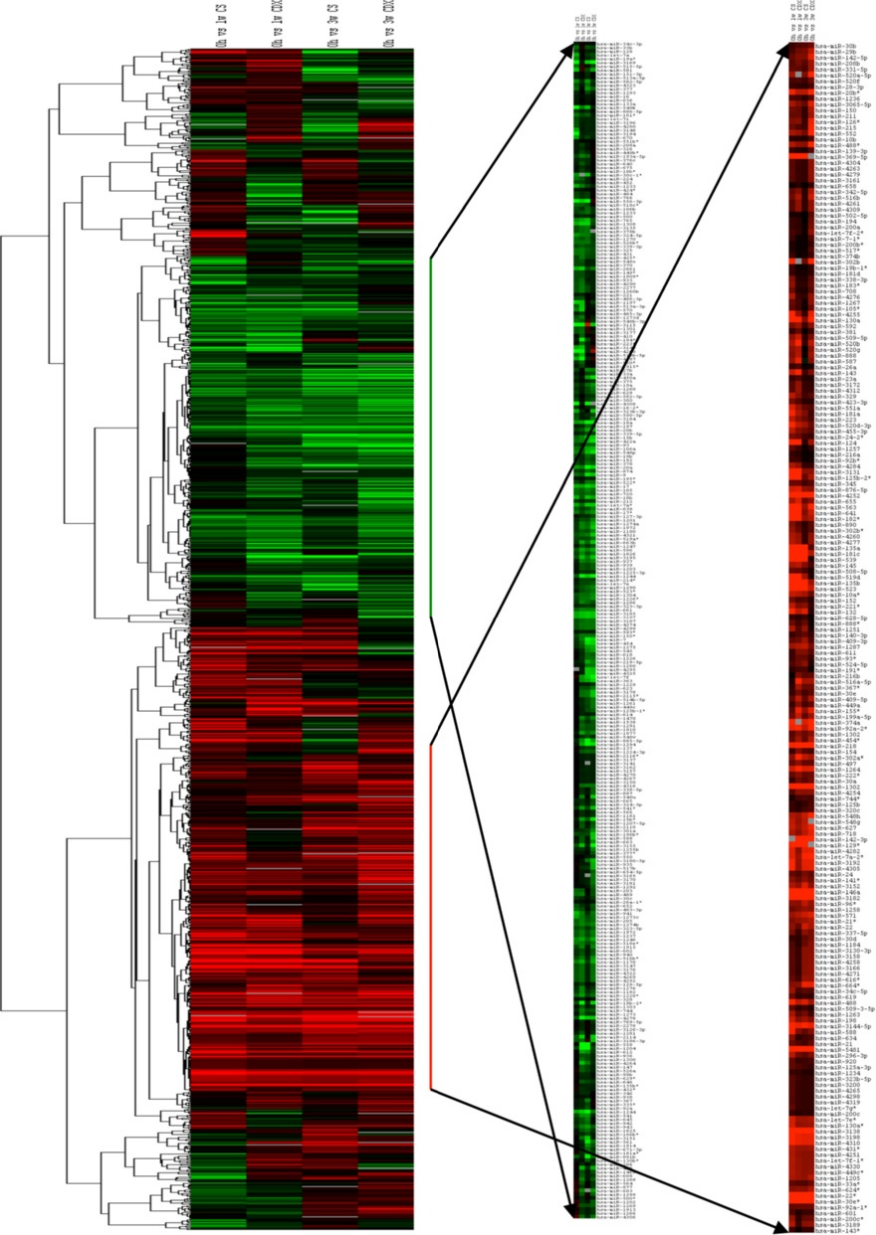
**Cluster analysis of fold change in expression of miRNAs in different treatment conditions.** Hierarchical clustering of log_2_ transformed FC expression of miRNA in four treatment groups, 1wk CSFBS, 1wk CDX, 3wks CSFBS and 3wks CDX (Table 1). Red line and green lines showing the expression patterns of 197 up regulated and 307 down regulated miRNAs (Supplemental data 3), respectively.

**Figure 3:**
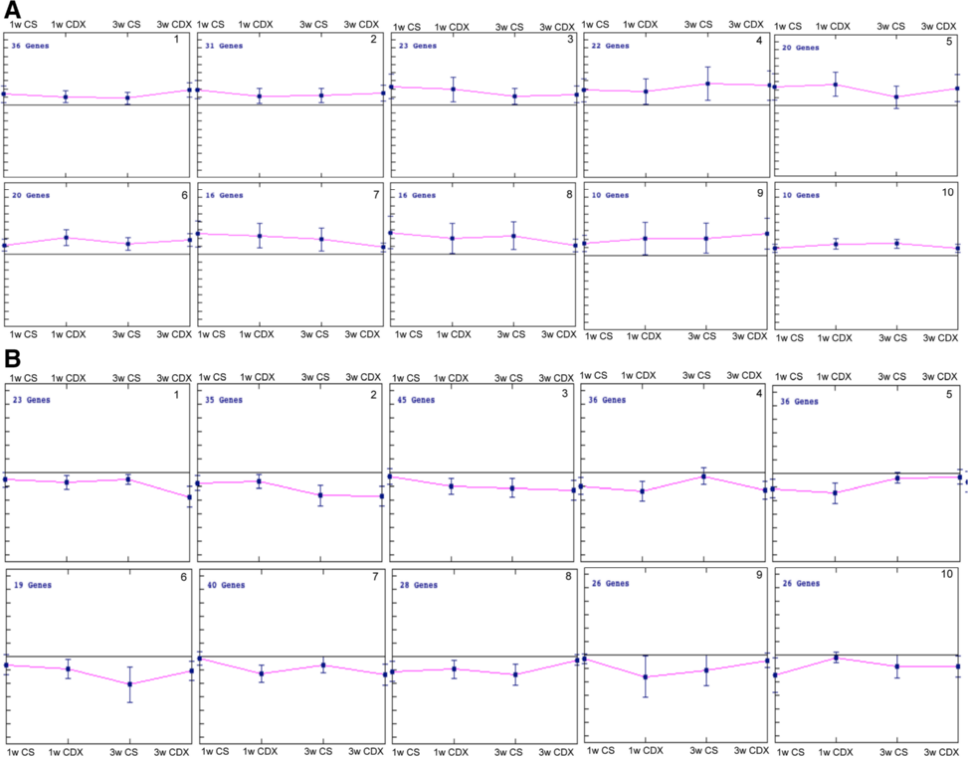
**K-median cluster analysis for differentially expressed miRNAs at different treatment conditions and time points. A)** Up regulated miRNAs separated into 10 clusters based on similar trends in expression patterns are depicted. MiRNAs in each cluster are showing a unique pattern of expression among 1wk and 3wks treatments of both CDX and CSFBS. **B)** Down regulated miRNAs divided into 10 clusters based on similar trends in expression are shown. MiRNAs in each cluster are showing unique patterns of expression during 1wk and 3wks treatments of CDX and CSFBS. Data shows median ± SD of expression of miRNAs in each cluster.

Clustering analyses using log_2_ transformed fold change (FC) values of four treatment conditions compared to -104S untreated cells showed two distinct clusters of up and down regulated miRNAs, which includes 307 down regulated and 197 up regulated miRNAs (Figure 2 and Additional file 6: Table S3). K-median clustering for the up-regulated miRNAs showed a trend of gradual increase in median FC in miRNAs in some clusters (cluster 1, 4 and 9) and a gradual decrease in some clusters (clusters 3, 7 and 8) from 1 week to 3 weeks treatments (Figure 3A and Additional file 7: Table S4). In down regulated profile there is also a trend of gradual decrease in median expression of miRNAs in some clusters (clusters 1, 2, 3 and 7) (Figure 3B and Additional file 7: Table S4). Of these lists, 100 miRNAs were chosen based on fold change and/or the z score ≥3.0 or ≤ -3.0 for validation using qPCR.

### Validated expression of miRNAs revealed distinct differences in expression in different treatment conditions

**Figure 4:**
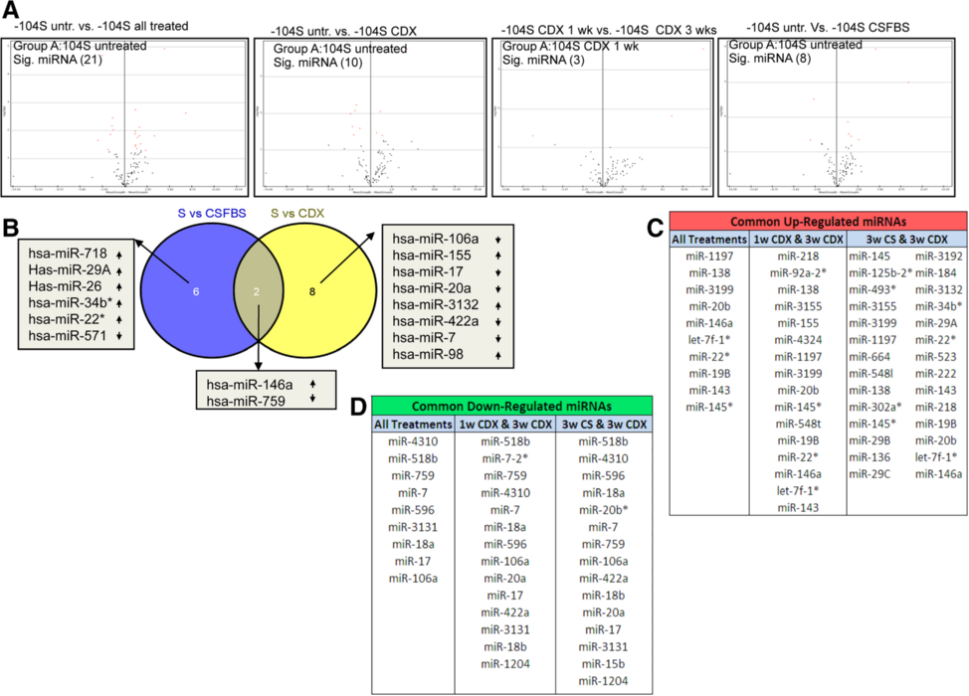
**Comparative analysis of the expression of validated miRNAs during progression to ADT and CDX resistance. A)** Volcano plots depicting significant miRNAs identified by two sample t-test in all treatment conditions compared to untreated -104S cells (1st panel from left), in all CDX treated cells compared to untreated S cells (2nd panel from left), in 3wks CDX treated cells compared to 1wk CDX treated S cells (3rd panel from left) and in all CSFBS treated cells compared to untreated S cells (4th panel from the left). **B)** Venn diagram using significant miRNAs identified in two sample t-tests (volcano plots) showing common and unique miRNAs between all -104S CSFBS and -104S CDX treated cells. **C**: List of common up-regulated miRNAs as noted in Venn diagram in all treatment conditions, between 1wk CDX and 3wks CDX and between 3wks CDX and 3wks CSFBS cells. **D**. List of down regulated miRNAs in all treatment conditions, between 1wk CDX and 3wks CDX and between 3wks CDX and 3wks CSFBS cells.

Analysis of qPCR data indicated a variable expression profile of a subset of miRNAs in LNCaP cells exposed to CDX and/or androgen withdrawal (CSFBS) for different time periods. Two sample Welch t-test with a *p*-value < 0.05 showed a significant change in expression of 21 miRNAs in treated samples compared to the untreated -104S cells (Figure 4A and Additional file 8: Table S5). Comparison between -104S untreated and either CDX or CSFBS treated samples identified 10 and 8 significant miRNAs respectively (Figure 4A) Comparative expression analysis between CDX 1wk and CDX 3wks also revealed significant change in expression in 3 miRNAs (Figure 4A). Venn Diagram of miRNA expression profiles following CDX treatment and androgen withdrawal indicated two common miRNAs (miR-146a and miR-759) but treatment specific differential expression was observed with 8 and 6 unique miRNAs, in CDX and CSFBS treated respectively (Figure 4B). Analysis of the up-regulated miRNAs revealed 10 miRNAs that showed 2-fold or higher expressions in all treatment conditions and time points (Figure 4C). There are also 30 miRNAs that showed 2-fold or higher expression in more than one treatment conditions. In the down regulated list, 9 miRNAs showed at least 2-fold reduction in expression in all treatment conditions and 15 miRNAs with ≥2.0-fold down regulation in more than one treatment conditions (Figure 4D).

**Figure 5:**
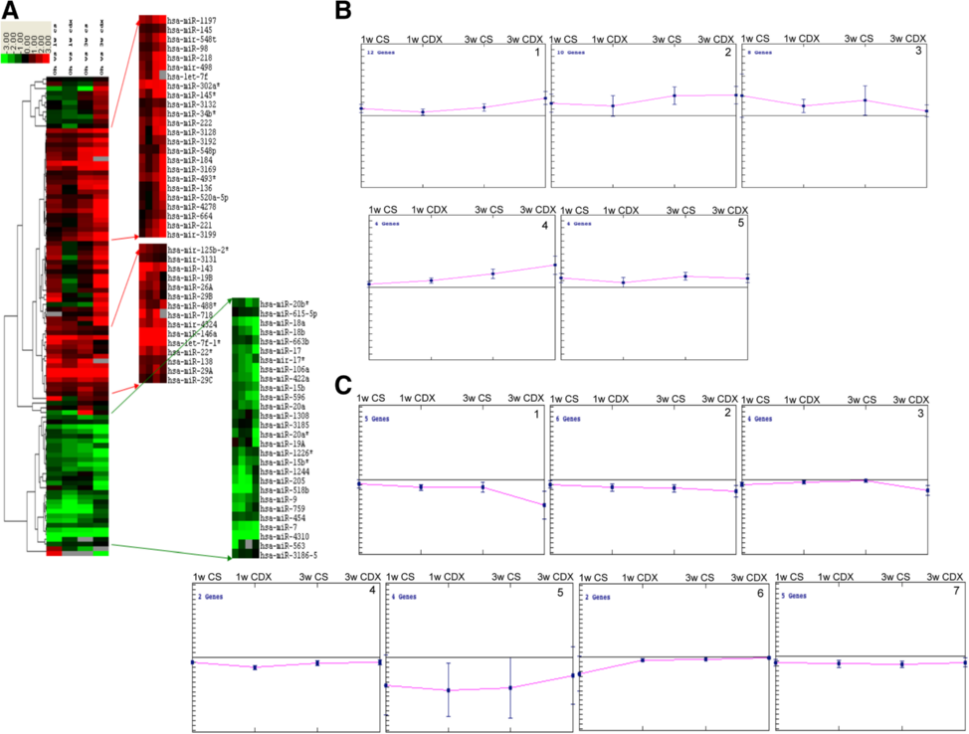
**Cluster analysis of fold change in expression of validated miRNAs in different treatment conditions. A)** Hierarchical clustering of log_2_ transformed FC expression of miRNA in four treatment groups, 1wk CSFBS, 1wk CDX, 3wks CSFBS and 3wks CDX (Table 1). Red arrows and green arrows showing the expression patterns of 21 up regulated and 22 down regulated miRNAs (Tables 2 and 3), respectively. **B**, **C)** K-median cluster analysis of the validated miRNAs in different treatment conditions **B)** Up-regulated miRNAs are divided into 5 clusters, which show unique trends of expressions. All miRNAs in each cluster show similar trends in expression in all treatment conditions and time points. **C)** Down regulated miRNAS are divided into 7 clusters each showing unique trend of expression pattern of miRNAs. All miRNAs in each cluster is showing similar patterns of expression. Data shows median ± SD of expression of miRNAs in each cluster.

Cluster analysis of log_2_ transformed FC in expression of 100 miRNAs showed three distinct clusters displaying common expression changes in 2 time points of two eatment conditions. Two clusters contained miRNAs with increased expression and one cluster containing down regulated miRNAs (Figure 5A and Additional file 9: Table S6). K-median clustering of the up-regulated miRNAs showed a distinct trend of gradual increase (clusters 1 [32%], 2 [26%] and 4[11%]) and gradual decrease (cluster 3 14%) in FC expressions as the treatment progressed (Figure 5B and Additional file 10: Table S7). Some of the miRNAs (21) showed across the board up regulations (Table 2). Similarly, in the list of down-regulated miRNAs a gradual decrease in expression (clusters 1[18%] and 2 [21%]) could be noted as the treatment progressed (Figure 5C) and a subset of miRNAs (22) showed down-regulation in all treatment conditions (Table 3). When compared with published expression database there are supporting reports on expression patterns of specific miRNAs in different types of cancer (Tables 2 and 3). There are also opposing reports on expression of miRNAs, which includes, miR-106a [25], miR-15b [26], miR-17 [27], miR-18 [28], miR-205 [29], miR-20 [30] and miR-7 [31] for down regulated miRNAs; and let-7f-1 [32], miR-136a [33], miR-143 [34], miR-146a [35], miR-218 [36], miR-22 [37], miR-222 [38] miR-29a [39], miR-34b [40], and miR-493 [41] for up-regulated miRNAs.

**Table 2:** Fold changes in expressions of up regulated miRNAs and its relevance with published reports

	**1wk CSFBS ∆ change**	**1wk CDX ∆ change**	**3wks CSFBS ∆ change**	**3wk CDX ∆ change**	**Expression in other Cancer**	**References**
hsa-let-7f-1	12.50	12.17	13.91	17.64		[42,43]
hsa-miR-136	3.69	3.44	5.16	4.30		[44,45]
hsa-miR-143	15.80	19.67	13.92	6.25		[46,47]
hsa-miR-146a	31.44	20.83	56.46	50.68		[48,49]
hsa-miR-218	4.64	2.33	18.44	11.49		[50,51]
hsa-miR-22	2.39	2.43	4.58	3.10		[52]
hsa-miR-22*	15.91	11.56	11.78	22.47		NA
hsa-miR-222	3.65	1.99	7.05	4.63		[53,54]
hsa-miR-29a	2.09	1.58	4.60	4.24		[55,56]
hsa-miR-302a*	2.97	7.73	3.87	4.48		[57,58]
hsa-miR-3138	6.53	5.94	6.66	5.96	-	NA
hsa-miR-3144b-5p	11.25	4.39	4.47	8.53		[59]
hsa-miR-3192	8.70	2.11	5.52	5.71	-	NA
hsa-miR-3199	1.25	4.54	2.71	6.76	-	NA
hsa-miR-34b*	3.15	2.34	6.69	8.64		[60,61]
hsa-miR-493*	1.31	1.42	2.31	15.06		[62]
hsa-miR-548h	2.12	3.76	4.06	6.72	-	NA
hsa-miR-548l	3.94	3.11	8.66	7.40	-	NA
hsa-miR-548p	3.88	2.44	4.64	1.55	-	NA
hsa-miR-548t	4.89	9.65	1.26	5.46	-	NA
hsa-miR-664	3.81	4.01	5.16	5.05		[63]

NA: Not available. *denotes the star strand, which is currently designated as -3p or -5p. Thickness of arrows represents number of publications available corresponding to the expression pattern.

**Table 3:** Fold changes in expressions of down regulated miRNAs and its relevance with published reports

	**1wk CSFBS ∆ change**	**1wk CDX ∆ change**	**3wks CSFBS ∆ change**	**3wk CDX ∆ change**	**Expression in other Cancer**	**References**
hsa-miR-106a	-2.37	-3.18	-5.29	-9.40		[64,65]
hsa-miR-1244	-3.40	-8.55	-5.14	-3.38	-	NA
hsa-miR-15b	-1.86	-2.02	-2.85	-3.33		[66,67]
hsa-miR-15b*	-2.42	-4.65	-3.83	-9.76		[68,69]
hsa-miR-17	-2.91	-3.37	-2.50	-24.99		[70,71]
hsa-miR-17*	-3.15	-3.13	-5.26	-3.94		[68,72]
hsa-miR-18a	-2.01	-4.36	-14.70	-14.13	-	[73]
hsa-miR-18b	-1.56	-2.28	-4.92	-21.36		[74,75]
hsa-miR-205	-6.33	-12.51	-12.06	3.89		[76,77]
hsa-miR-205*	-0.35	-1.34	-2.85	-3.23	-	NA
hsa-miR-20a	-2.73	-3.31	-3.78	-4.12		[71,75,78]
hsa-miR-20a*	-1.78	-1.25	-2.64	-18.17	-	[69,75]
hsa-miR-20b*	-0.64	-0.56	-7.99	-4.60	-	[74,75,79]
hsa-miR-3131	-2.35	-2.97	-3.14	-3.41	-	[80]
hsa-miR-3185	-2.95	-3.24	-4.17	-5.76	-	[80]
hsa-miR-422a	-3.15	-3.15	-4.92	-6.87	-	[81]
hsa-miR-454	-0.58	-1.89	-3.28	-3.76		[75,82]
hsa-miR-518b	-2.64	-6.42	-5.61	-12.23	-	[83]
hsa-miR-596	-2.45	-3.94	-15.83	-12.71	-	[84]
hsa-miR-759	-13.05	-10.92	-6.17	-5.86		[82]
hsa-miR-7	-3.00	-5.23	-6.76	-11.08	-	[85]
hsa-miR-9	-2.64	-4.35	-4.53	-3.55		[86]

NA: Not available, *denotes the star strand, which is currently designated as -3p or -5p. Thickness of arrows represents number of publications available corresponding to the expression pattern.

### Involvement of miRNAs in specific cellular processes which differ between treatment conditions

MicroRNAs exhibiting up regulation or down regulation in all treatment conditions compared to untreated -104S cells (Tables 2 and 3), were used for function and disease relevance to understand the possible alterations in the ellular processes as LNCaP cells progressed towards androgen withdrawal and AR antagonist resistance. We used RT-PCR FC values of the up regulated or down regulated miRNA for the analysis using IPA software (Ingenuity Systems). Based on -log (p-values) with -values <0.05, a percentage of miRNAs were assigned to certain disorder or cellular processes (Additional file 11: Figure S4). When the functional profiles of the p-regulated miRNAs were compared, there is a decrease in the percentage of miRNAs involved in cancer, dermatological disease, and hematological disease between 3wks and 1wk CDX treated cells whereas an increase in the percentage of miRNAs involved in endocrine system disease, gastrointestinal disease hepatic system disease, reproductive system disease and metabolic disease in 3wks CDX treated S cells compared to 1wk CDX treated cells (Additional file 11: Figure S4A). In cells subjected to androgen withdrawal, there are also differences in percentage of miRNAs in different cellular processes between 1wk and 3wks treatments, which show a reduction in hematological diseases, cellular development, and reproductive system disease. An increased involvement of miRNAs in a variety of cellular processes also was noted in 3wk CSFBS treated cells, which includes cell death and survival, cell movement, endocrine system disease, gastrointestinal disease, hematological disease, hepatic system disease and metabolic disease (Additional file 11: Figure S4A).

In the list of down-regulated miRNAs, there is an increase in percentage of miRNAs involved in cell morphology, cell movement, dermatological disease, gastrointestinal disease, renal urological disease, and reproductive system disease as cells progressed from 1wk to 3wks CDX treatment. A decreased percentage of down regulated miRNAs in specific cellular processes also could be noted in these cells, which includes cellular development, endocrine system disease, hepatic system disease, tumor morphology and inflammatory response. Androgen withdrawal for 1wk to 3wks also showed changes in percentage of miRNA involvement (Additional file 11: Figure S4B). An increased percentage of down regulated miRNAs involved in cancer, cell death and survival, dermatological disease, endocrine system disease, gastrointestinal disease, metabolic disease, and tumor morphology is noted in 3wks CSFBS treated cells. 3wks androgen withdrawal also showed a decrease in miRNA percentage involved in cell growth and proliferation, cell movement, cell-cell signaling, DNA replication and repair and renal-urological disease (Additional file 11: Figure S4B).

**Figure 6:**
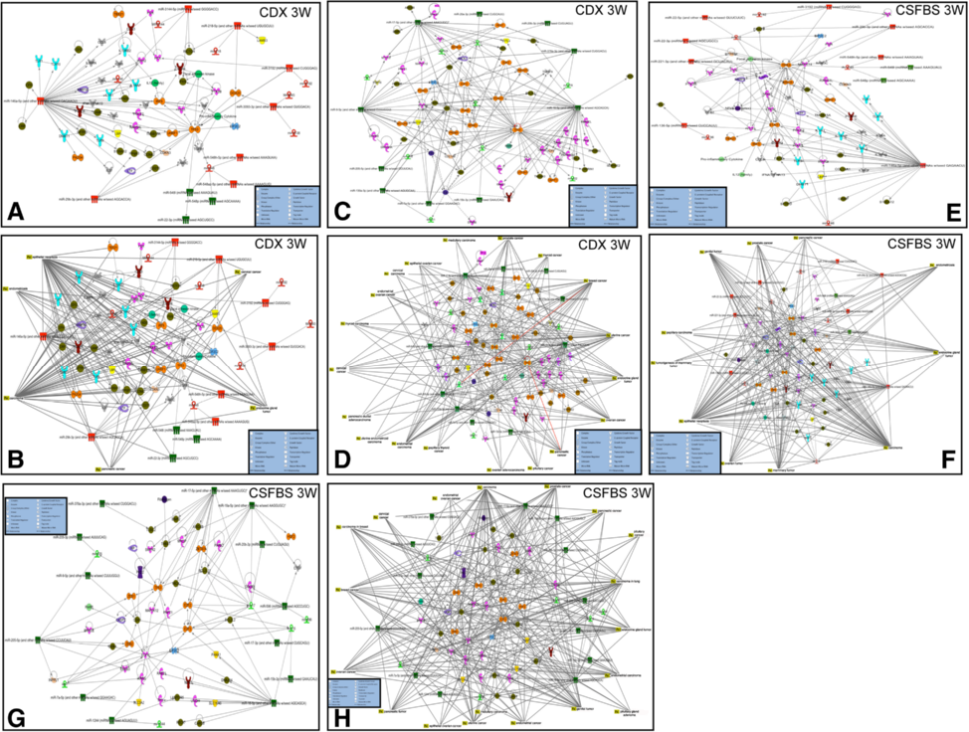
**Analysis of miRNA-regulated network of interacting partners involved various signaling pathways. A)** Log_2_ transformed FC values of 21 up regulated miRNAs were imported into the Core analysis tool of IPA software and a network for interacting miRNAs and their direct target proteins and proteins that target any of the up regulated miRNAs has been generated for 3wks CDX treated cells. Red symbols denote miRNAs or mature miRNAs. Proteins include growth factor/cytokines, enzymes, kinases, phosphatases, peptidases, translational regulator, transcriptional regulators, and transporters. **B)** The network in A is overlayed with disease relationship showing association of miRNA and protein interactions with various types of cancer. **C** and **D)** Regulatory network of the log_2_ transformed FC values of 22 down regulated miRNAs and their target proteins in 3wks CDX treated cells **(C)** and overlay of disease association of the miRNA-protein regulatory network **(D)**. Green symbols represent down regulated miRNAs and mature miRNAs. **E** and **F)** Regulatory network of log_2_ transformed FC values of the up regulated miRNAs and their target proteins in cells treated with CSFBS for 3wks **(E)** and an overlay of disease relationship with the regulatory network in E. Red symbols represent miRNAs and mature miRNAs. **G** and **H)** Regulatory network of log_2_ transformed FC values of the down regulated miRNAs and their target proteins in cells treated with CSFBS for 3wks **(G)** and an overlay of disease association with the regulatory network in G **(H)**. Green symbols represent down regulated miRNAs and mature miRNAs.

The *in silico* function analysis of the target miRNAs in different treatment conditions indicated a complex interaction of a network of miRNAs and their target proteins in these cells which rendered them adaptive to the androgen withdrawal and treatments with AR antagonists. Next, we analyzed the network of interactions among target miRNAs. We used the log_2_ transformed FC values of the subset of validated miRNA (Tables 2 and 3) for analysis of the functional interrelationship among miRNAs (Figure 6). The network for up-regulated miRNAs and its putative targets in 3wks CDX showed that miR-3192 directly and miR-218 indirectly through DEAD box protein DDX20 target p53. MiR-146a also directly regulates a number of Toll-like receptors (TLR1, TLR9 and TLR10), cytokine receptors and its associated proteins (IL1R1, IL12RB2, IL1RAP) and chemokine receptors (CXCR4, CCR3) (Figure 6A). In addition, miR-146a directly targets a number of growth factors and cytokines (Figure 6A). These miR-146a targets are also shown in the up-regulated network of 3wks CSFBS, in addition to BRCA, which is a direct target of miR-146a (Figure 6E). MiR-3192 targeting of p53 is also noted in 3wks CSFBS but not of miR-218 (Figure 6E). As noted in the networks, p53 also regulates a number of miRNAs, such as, miR-29b-3p, miR-22-3p, miR-22-5p and miR-221-3p (Figure 6A and E). When the networks are overlaid with diseases, a number of miRNAs in the up-regulated list of both 3wks CDX and 3wks CSFBS showed involvement in a variety of cancers including pancreatic cancer, endocrine gland tumor, prostate cancer, ovarian tumor, mammary tumor, cervical cancer and epithelial neoplasia (Figure 6B and F).

In the list of down-regulated miRNAs there is direct targeting of E2F2, E2F3, MAP3K12, FXR1 and AR interacting nuclear protein kinase HIPK3 by miR-17-5p. MiR-16p also directly targets BCL2L2, cell death suppressor BNIP2, EGFR, RAB21 and single strand purine rich DNA binding protein PURA (Figure 6C and G) in both 3wks CDX and 3wks CSFBS treated cells. Also a number of miRNAs in the down regulated list down regulate a number of common targets, such as E2F3 is targeted by miR-1244, miR-596, miR-18a-5p, miR-16-5p and miR-17-5p. EGFR is also targeted by miR-7a-5p and miR-16-5p. Overlay of diseases on networks revealed a distinct association of miR-17-5p, with various cancers including prostate cancer, breast cancer and pancreatic cancer and endocrine gland tumors. An association of miR-16-5p with prostate cancer, pancreatic cancer, pituitary cancer and endometrial cancer also could be noted (Figure 6D and H). These networks indicate a complex interplay of microRNAs and proteins during development of resistance of LNCaP cells to androgen withdrawal and treatment with CDX.

### Target identification of the subset of miRNAs revealed potential activation and/or inactivation of a number of proteins involved in different signaling networks

**Figure 7:**
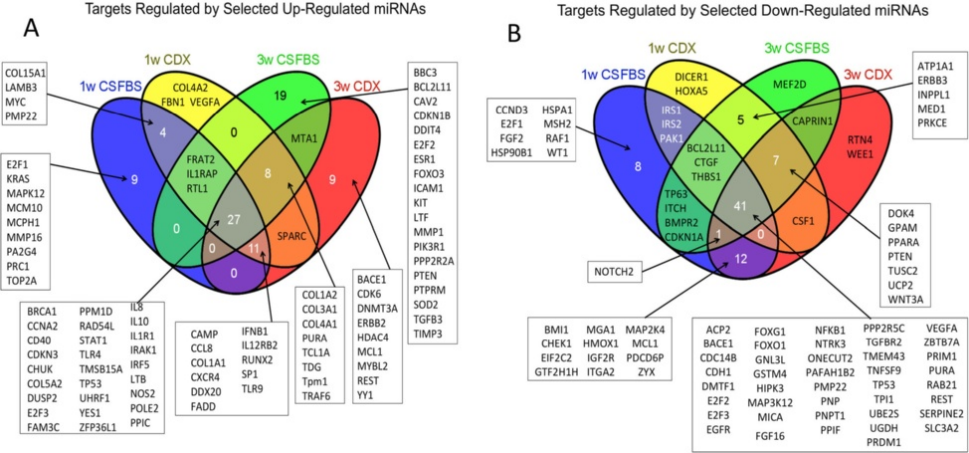
**Analysis of treatment specific targets regulated by validated miRNAs. A)** Venn diagram showing specific targets that are potentially regulated by miRNAs that are up regulated in different treatment conditions. Putative targets were identified based on the regulatory network generated by IPA software. **B)** Venn diagram depicting predicted targets regulated by the down-regulated miRNAs in different treatment conditions. Targets were identified based on the regulatory network using IPA software.

A total of 21 up regulated and 22 down regulated miRNAs in all treatment conditions were used for identification of protein targets using IPA, miRDB and TargetScan software. Venn diagram of the putative targets of up-regulated miRNAs derived from the network generated using the IPA software showed 27 proteins that are common in all treatment conditions (Figure 7A). A number of which are known tumor suppressors such as BRCA1[87], TP53 [88], RAD54L [89], IRF5 [90], DUSP2 [91], IKK1 or CHUK [92]. Other targets of up regulated miRNAs in different treatment conditions also includes proteins that inhibit tumor progression, such as DNMT3A [93], FADD [94], pTEN [95], FOXO3[96], DDX20 [97] and PA2G4 (EBP1)[98]. There are also 41 common targets of the down-regulated miRNAs in all treatment conditions (Figure 7B). Some of the targets are known oncoproteins, which includes EGFR [99], VEGFA [100], ZBTB7 (POKEMON) [101], acid phosphatase 2(ACP2) [102] and NFKB1 [103]. Additionally, proteins that are overexpressed in cancer cells are among the targets of down-regulated miRNAs in different treatment conditions, which includes Wnt3A [104], PPARα [105], UCP2 [106], CSF1 [107], and MED1 [108].

### Targets of miRNAs showed the predicted expression profiles in LNCaP cells subjected to androgen withdrawal and CDX treatment

**Figure 8:**
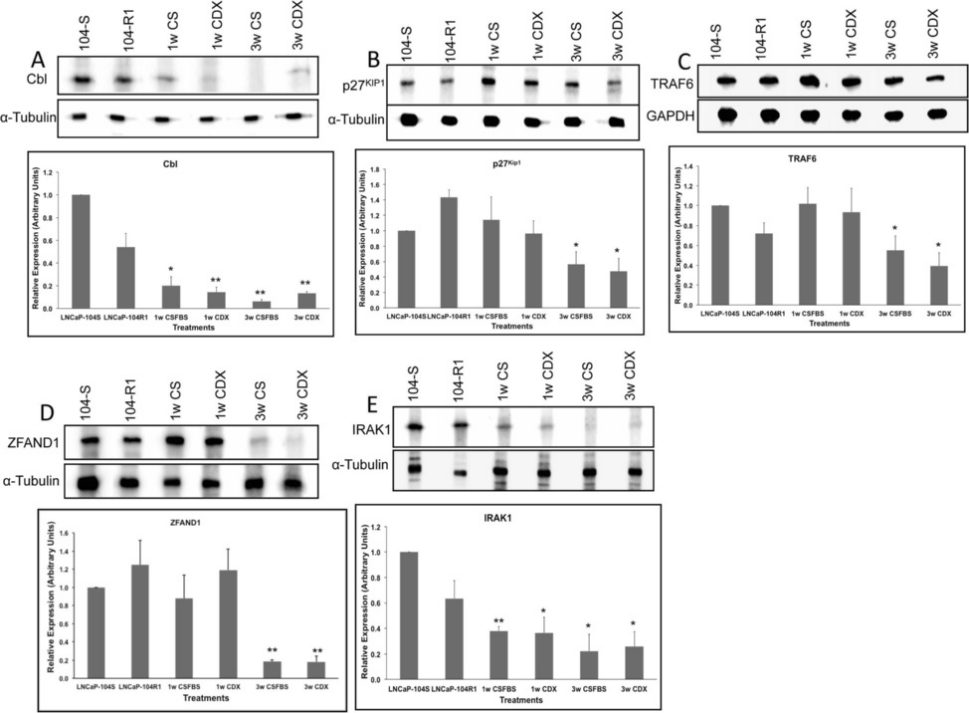
**Comparative analysis of target protein expression in LNCaP sublines in different treatment conditions.** Western blot analysis of the target proteins identified using TargetScan and miRDB online prediction tools for a particular miRNA (shown in Table 2). Total proteins from -104S cells untreated and treated for different times, and -104R1 cells untreated were used for immunoblotting using anti-cbl **(A)**, -p21Kip1 **(B)**, -TRAF6 **(C)**, -ZFAND1 **(D)** and –IRAK1 **(E)** antibodies. Upper panel shows representative images of the western blots. Bottom panel shows densitometric analysis of relation protein expression. Data shows mean ± SD of at least three independent experiments. *p < 0.05, **p < 0.01, significance was calculated between LNCaP-104S untreated vs. specified treated cells.

**Figure 9:**
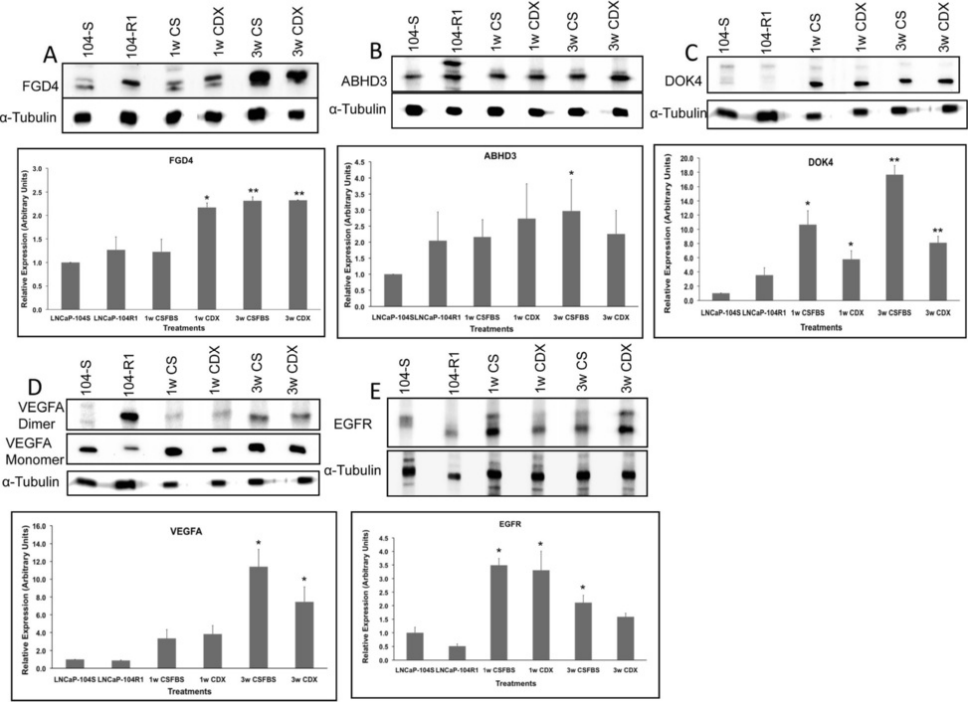
**Comparative analysis of target protein expression in LNCaP sublines in different treatment conditions.** Western blot analysis of predicted target proteins regulated by down regulated miRNAs (shown in Table 3). Total proteins from all treated and untreated cells were used for immunoblot analysis using anti- FGD4 **(A)**, ABHD3 **(B)**, -DOK4 **(C)**, VEGFA **(D)**, and EGFR **(E)** antibodies. Upper panel shows representative images of the western blots. Bottom panel shows densitometric analysis of relation protein expression. Data shows mean ± SD of at least three independent experiments. *p < 0.05, **p < 0.01, significance was calculated between LNCaP-104S untreated vs. specified treated cells.

Further identification of the targets of selected miRNA (Table 2) was conducted by miRDB and TargetScan database search and proteins that are regulated by one or multiple miRNAs from our list and received higher target scores in either or both searches are presented in Tables 4 and 5. The selected proteins that are regulated by the up-regulated miRNAs include TNF receptor associated factor 6 (TRAF6) [109], interleukin receptor associated kinase1 (IRAK1), BCL6 co-repressor-like 1(BCORL1) [110], Cbl [109], neuro oncological ventral antigen1 (NOVA1) [111], coiled coil domain containing 67 (CCDC67) [112] NET1 [113], ZFAND1(Acc.# NM_024699), RGS6 [114] CDKN1B (p27Kip1) [115], IGFBP5 [116] and RNASEL [117] (Table 4). The proteins that are potentially regulated by the down-regulated miRNAs include, ABHD3 [118], FGD4 [119], CCNJ [120], CHAMP1 [121], Myb [122], PIK3CD [85], VEGFA [123], SPOPL [124], RAB9B [125], EGFR [126], E2F1 [127] and DOK4 [128]. Western blot analysis confirmed the predicted expression profiles of some of the targets. Expression of Cbl, p27Kip1, TRAF6, IRAK1, and ZFAND1 were significantly and progressively down regulated in cells treated with CDX or subjected to androgen deprivation (Figure 8). Similarly, expression of FGD4, VEGFA, EGFR, DOK4 and ABHD3 were up regulated to moderate to high levels in cells treated with CDX or CSFBS (Figure 9).

**Table 4:** Predicted mRNA Targets of Up-Regulated miRNAs

**Gene ID**	**Gene description**	**miRNAs**	**miRDB (target score)**	**TargetScan (Context + Percentile)**
**TRAF6**	TNF receptor-associated factor 6, E3 ubiquitin protein ligase	miR-146a	100	94,94,65,50,38,19
**IRAK1**	Interleukin receptor associated kinase 1	miR-146a	87	98
**BCORL1**	BCL6 corepressor-like 1	miR-146a, miR-143, miR-548t	77,68	98,80
**NOVA-1**	Neuro-oncological ventral antigen1	miR-146a, miR-143, miR-548l	97,93,86	97,89,87
**CCDC67**	Coiled-coil domain containing 67	miR-22	77	99,97
**Cbl**	Cbl proto-oncogene, E3 ubiquitin protein ligase	miR-22, miR-222,miR-136, miR-3199,miR-1197	87,83,64,55	86,84,75,61,43,33,32,31
**NET1**	Neuroepithelial cell transforming 1	miR-22, miR-222,miR-143	93,61	98
**ZFAND1**	Zinc finger, AN1-type domain 1	miR-548h, miR-548l,has-miR-548t,miR-136	96,70	93,83,99,52
**RGS6**	Regulator of G-protein signaling 6	miR-222	75	91,96,12
**CDKN1B**	Cyclin-dependent kinase inhibitor 1B (p27, Kip1)	miR-222	85	93,95
**IGFBP5**	Insulin-like growth factor binding protein 5	miR-143	N/A	99
**RNASEL**	Ribonuclease L	miR-146a, miR-548l	74	81

**Table 5:** Predicted mRNA Targets of Down-Regulated miRNAs

**GeneID**	**Gene Description**	**miRNAs**	**miRDB (target score)**	**TargetScan (Context + Percentile)**
**ABHD3**	Abhydrolase domain containing 3	miR-1244, miR-130a, miR-205	74, > 85	84
**CCNJ**	Cyclin J	miR-205-5p	90	98
**CHAMP1/ZNF828**	Chromosome alignment maintaining phosphoprotein 1	miR-7-5p, miR-378a-3p	60-96	96
**MYB**	v-Myb myeloblastosis viral oncogene homolog (avian)	miR-15b-5p, miR-16-5p	74	96
**PIK3CD**	Phosphoinositide-3-kinase, catalytic, delta polypeptide	miR-7-5p	94	91
**VEGFA**	Vascular endothelial growth factor A	miR-205, miR-15b	79	97
**FGD4**	RhoGEF and PH domain containing 4	miR-17, miR-106a, miR-20a	100	98
**SPOPL**	Speckle-type POZ protein-like	miR-9-3p	97	97
**RAB9B**	RAB9B, member RAS oncogene family	miR-15b-5p, miR-16-5p, miR-130a-3P, miR-9-5p	67-86	67-86
**EGFR**	Epidermal growth factor receptor	miR-7	81	93
**E2F1**	E2F transcription factor 1	miR-205, miR-17, miR-20b	77	80
**DOK4/IRS-5**	Docking protein 4	miR-205	N/A	94

## Discussion

Our studies on profiling and validation of miRNA expressions during transition of AD LNCaP-104S cells to AI and CDX resistant cells revealed activation and inactivation of several signaling networks. We noted a difference in miRNA expressions between the AI subline LNCaP-104R1, and freshly generated CDX resistant LNCaP-104S cells, which includes some of the up regulated (miR-146a) and down-regulated miRNAs (miR-15b-3p and miR-18b). Although we noted differential expression of miRNAs between CS-FBS and CDX treated LNCaP cells we selected only miRNAs that showed either up regulation or down regulation in all treated samples for analysis of their putative targets. Despite similar expression profile of specific miRNAs in CS-FBS and CDX treated samples, some the targets such as DOK4 and VEGF showed differential expression in CSFBS and CDX treated cells. Presumably, this could be the effect of regulation of multiple targets by a given miRNA, which may indirectly affect the net expression of DOK4 and VEGF.

Over expression of miR-146a was noted in all treated cells contrary to the study showing loss of expression of miR-146a in CRPC [129]. Increased miR-146a expression was substantiated by down regulation of its two bona fide targets TRAF6 and IRAK1 in both -104R1 and 3wks treated -104S cells. MiR-146a expression is induced by NF-κB [130] and acts in a negative feedback loop through degradation of TRAF6 and IRAK1 to reduce NF-κB signaling and inflammatory response. An increase in transcription of miR-146a, as a result of elevated NF-κB activity is noted in thyroid cancer [131] and down regulation of miR-146a is associated with hyperactivation of NF-kB [132]. Despite this association, an increase in NF-kB1 expression could be predicted in treated -104S cells, as NF-kB1 is a direct target of the down-regulated miRNA miR-9 [133]. Increased expression of the other subunit RelA, which heterodimerizes with NF-kB1 also could be predicted as it is a direct target of the down-regulated miR-7 [134]. It appears that the NF-kB signaling pathway is activated in the early stages of gaining resistance to CDX/androgen blockade and the increased expression of miR-146a is a secondary effect of the activation of the NFkB pathway. As a result, in the initial stages of anti-androgen drug resistance there is decreased inflammatory response but down regulation of tumor suppressor targets of miR-146a, BCORL1 [135] and RNASEL [136], which may not be detected in fully developed CRPC.

The EGFR signaling pathway could be activated also in the early stages of androgen blockade and CDX treatment. The evidence of EGFR pathway activation in the treated -104S cells is from our results showing an increased expression of EGFR, down regulation of miR-7 and up-regulation miR-222, which are the miRNA regulators of EGFR. Decreased expression of p27Kip1 and Cbl, as two other targets of the up-regulated miR-222, further aid activation of EGFR signaling. C-Cbl, an E3 ubiquitin ligase, inactivates ligand-bound EGFR through EGFR-Cbl complex formation leading to its degradation [137]. C-Cbl activation mediates the tumor suppressive effects of EPhB6 and inhibits cancer cell invasiveness [138]. C-Cbl is also targeted by the up regulated miRNA miR-136 in all treated cells. Down regulation of c-Cbl in treated -104S and untreated -104R1 cells possibly promotes antiandrogen resistance through EGFR stabilization. A loss of expression of AR was noted in these cells (data not shown), which supports the report showing an inverse relationship between expression of AR and EGFR in prostate cancer patients [139]. Up regulation of miR-136 in treated -104S cells was substantiated by the loss of expression of the miR-136 target ZFAND1, an uncharacterized AN1 type zinc finger domain 1 containing protein.

Activation of PI3K/AKT signaling axis also could be predicted in treated -104S cells, as a result of down regulation of miR-7, which inhibits tumor growth and metastasis through inhibition of PI3K/AKT pathways [85,140]. Down regulation of miR-7 in cancer cells including glioblastoma and its inhibitory effects on EMT and metastasis is well documented [141]. Activation of this pathway could be further aided by over expression of MiR-22 in all treated cells, which exerts a proto oncogenic effect through down regulation of PTEN in AI prostate cancer cells [142]. Additionally, activation of VEGF and DOK4 could be predicted, as these proteins are over expressed in treated -104S cells possibly as a result of down regulation of their regulatory miRNA, miR-205. Earlier studies showed an association between poor prognosis of localized prostate cancer and epigenetic repression of miR-205 [77], and thus confirms the relevance of the loss of miR-205 in development of CDX resistance. DOK4 is a newly identified substrate of ligand-bound insulin receptor (IRS-5), which upon phosphorylation translocates to mitochondria and recruits c-Src kinase to the mitochondria. Up regulation of DOK4 is also noted in renal cell carcinoma [128]. VEGFA is a target of miR-15b-5p also, which showed 2-10-fold reduction in expression in treated -104S cells and in chemotherapy-resistant squamous cell carcinoma [67].

Other than modulation of specific signaling axis, altered expression of miRNA clusters is also evident in our study. Members of the miR-17-92 and its paralogous miR-106a-363 clusters, miR-17, miR-18a, miR-18b, miR-20a and miR-106a showed ~9-10-fold down regulation upon CDX treatment and androgen blockade. In support of our observation, loss of expression of miR-17 [71], miR-18a [73], miR-20a [78] and miR-106a [65] are reported in breast and other cancers. Loss of expression of miR-106a, miR-17 and miR-20a are further supported by an increased expression of their target protein FGD4 in these cells. Contrary to the published study [143], over expression of miR-34b was noted in AI and CDXR cells, however, an inverse relationship between miR-34b expression and disease free survival of triple negative breast cancer has been reported [61], which suggests that miR-34b expression may be dependent on the status of hormone responsiveness. Among the other up regulated miRNAs, over expression of let-7f1 [42], miR-143 [46], miR-218 [50], miR-29a [55], miR-302a [57], miR-3144 [59], miR-493 [62] and miR-664 [63] in cancer cells has been reported earlier. Our study also identified a number of miRNAs with > 2-fold difference in expression such as miR-3138, miR-3192, miR-3199, and a subset of miR-548 series, which are not yet known to be involved in development of CRPC.

Additional miRNAs such as miR-518b, miR-205 and miR-596 showed > 10-fold loss of expression upon CDX treatment or androgen withdrawal. In support of our observation, loss of expression and tumor suppressor functions of mir-518b and miR-596 has been documented in other cancers [83]. MiR-1244 and miR-759 are two other miRNAs that are significantly down regulated in all treated cells. This is substantiated by an increased expression of their common target ABHD3 in these cells [118]. Among the other down regulated miRNAs, miR-9 and miR-422a are known to have tumor suppressor roles in various cancer cells [81,86], whereas miRNAs -454, -3131 and -3185 are noted for the first time to be deregulated during progression of CRPC.

Functional contribution of some of the identified microRNAs in development of CRPC has been previously reported. Over expression of miR-222/221 in AI LAPC4 cells was shown to promote androgen independent cell growth, which was abrogated upon expression of anti-miR-222/221 inhibitors [21]. Down regulation of miR-205 has been correlated with advanced prostate cancer and ectopic expression of miR-205 suppressed AR and MAPK signaling and inhibited cell growth [144]. Down regulation of miR-17 in AI prostate cancer cells also has been demonstrated. Expression of pre-miR-17 in these cells prevented AR induced gene transcription and inhibited cell proliferation [145].

Analysis of the altered cellular processes during progression towards CDX resistance and androgen independence showed a decreased percentage of miRNAs involved in cancer but an increased percentage in reproductive system, endocrine system, hepatic system and metabolic diseases. It can be speculated that up regulated oncomirs at earlier stages aid in transformation of cells through suppression of tumor suppressors. Whereas, at later stages accumulation of abnormal cellular events triggers expression of additional sets of miRNA, which inhibit key regulatory proteins involved in metabolic process, hormone response and other cellular events. Our qRT-PCR FC data indicate differential expression of miRNAs between 1wk and 3wks treatment, which would have been undetected had the profiling been done only in CDX sensitive/AD and –resistant/AI cells. In silico analysis identified a number of targets that are potentially regulated by one or more altered miRNAs. This includes, two mitosis regulatory proteins CCNJ and CHAMP1 (ZNF828) [120,121], two oncogenic proteins PIK3CD [85] and MYB that are over expressed in CRPC [122], a protein trafficking regulatory protein RAB9B, a ubiquitination promoting protein SPOPL involved in the Hedgehog/Gli signaling pathway [124] and E2F1 transcription factor [127].

In summary, our results and in silico network analysis suggest that inhibition of expression of TP53, BRCA, Toll like receptors, IRAK1, STAT1, CHUK and FADD by the up regulated miRNAs, and increased synthesis of EGFR, NFkB1/RelA, E2F family members, BCL2L2, ZBTB7A, EGO2 (EIF2C2) and ZEB2 as the targets of down regulated miRNAs are part of the events that support growth and survival of AI LNCaP cells. During treatment with AR antagonist in androgen-deprived condition, additional inhibition of expression of DDX20, URF1, IRF5 and CDKN3 as the targets of the up regulated miRNAs and increased expression of PRDM1, DOK4, TNFSF9 and NOTCH2 as a result of down regulated miRNAs may provide additional protection against CDX induced cell death. In-depth studies are needed to accurately determine the activation and inactivation of specific signaling pathways during development of insensitivities of prostate cancer cells to AR antagonistic drugs.

## Conclusion

The overall evaluation of the changes in expression profiles of miRNAs during transition of CDX sensitive and AD cells to the CDX resistant and AI ones demonstrates that not any one or two miRNAs are responsible for development of drug-resistant prostate cancer. Instead, a complex network of activation and inactivation of specific signaling pathways aided by degradation or accumulation of the target mRNAs as a result of differential expression of a significant number of miRNAs plays the pivotal role. Also, there are transient changes in the expression of miRNAs as well as their target proteins during transition of cells towards ADT resistance, which may provide growth and survival advantage to a subset of cancer cells; and these changes in miRNA/mRNA signature may be missed in already developed castration resistant prostate cancer. This study provides a predictive tool for monitoring the susceptibility of development of anti-androgen therapy resistant prostate cancer.

## Materials and methods

### Cell culture, and treatments

The androgen responsive LNCaP-104S and androgen independent LNCaP-104R1 cells were generous gifts from Dr. Shutsung Liao, University of Chicago. These LNCaP sublines were isolated and characterized as previously described [146]. LNCaP-104S cells were maintained in DMEM (Gibco) containing 10% FBS (Atlanta Biologicals), 1nM DHT (Sigma-Aldrich), 1% Antibiotic/Antimycotic (Invitrogen). LNCaP-104R1 cells were maintained in DMEM containing 10% charcoal-stripped FBS (CSFBS), 1% Antibiotic/Antimycotic. Isolation of androgen independent cells was conducted by passaging LNCaP-104S cells in DMEM/10% CSFBS, supplemented with or without 5 μM Bicalutamide (Fluka) (Casodex, CDX) for 3 weeks (detail treatment method is in the Additional file 12 supplemental method section). Cells were harvested for protein or RNA extraction at 7 and 21 days post treatment.

### RNA extraction and cDNA synthesis

Total RNA was extracted from untreated and treated cells using the Cell-to-Cts kit (System Biosciences) according to the manufacturer’s instructions. Total RNA was converted to cDNA utilizing the QuantiMir RT Kit (System Biosciences) according to the manufacturer’s instructions. Briefly, small RNAs were first tagged with polyA-tails; oligo-dT primers were annealed next, and converted to cDNA by reverse transcription.

### Quantitative real-time PCR

Expression of mature miRNAs in untreated and treated LNCaP cells was determined by quantitative real-time PCR (qRT-PCR) using the miRNome microRNA Profiling Kit (System Biosciences) and cDNAs according to the manufacturer’s instruction. The kit provides specific primers for 1,113 mature miRNAs and 3 internal control snRNAs. MiRNA IDs listed in the text are based on Sanger miRBase identifiers. Primers were designed to maintain uniform amplification efficiencies. qRT-PCR was conducted using the Applied Biosystems 7900HT thermal cycler and data analyzed using and SDS2.3 software. DNA concentrations were reported through SYBR Green fluorescence and normalized to that of the passive reference dye, ROX.

### Statistical analysis of miRNA expression

Ct values generated by the SDS2.3 software were normalized according to the average Ct values of the three internal controls provided with the miRNome Profiler kit using qbasePLUS software (Biogazelle). In order to ensure the integrity of the ∆Ct values, we utilized the Genorm software (Biogazelle) to identify 7 additional miRNAs displaying stable expressions between samples. The Ct values of all miRNAs were then normalized to these 10 controls. The relative expression values were then generated using qbasePLUS software and used in additional analysis (detail explanation is in the Additional file 12 supplemental method section). The Ct values were used to derive ΔΔCt values using the miRNome analysis software (SBI). Candidate miRNAs with higher fold change values in each treatment conditions were determined by z score calculation as described in the Additional file 12 supplemental method section.

Clustering was performed using log_2_ transformed average ΔΔCt values and the Cluster 3.0 software (Michiel de Hoon, Univ of Tokyo, based on Eisen Lab Cluster software). Hierarchical clustering of the miRNAs was based on the average linkage of the Pearson’s correlation values. The relative expression values were Log_2_ transformed and used for evaluation of differences among groups of treated and untreated cells by performing a Welch t-test using MultExperiment Viewer (MEV) software. Results of the t-test were displayed in Volcano plots using -log_10_ P values of the log_2_ transformed values. The K-median clustering of the normalized values were performed using MEV software.

Next, identification of target mRNAs, creation of miRNA-protein networks, and identification of altered cellular processes were conducted using the IPA software (Ingenuity Systems). The ∆∆Ct values were used for a core analysis by selecting all tissue and cell types and using a stringent filter and generating direct relationships only. From the core analysis, mRNA targets were identified for each sample and these mRNAs were used to generate the Venn diagrams using online tool (Venny: http://bioinfogp.cnb.csic.es/tools/venny/index.html). The miRNA-protein network generated by the core analysis was overlayed with different cancer types and members of the network that displayed alterations in those cancers were identified using the Interactive Pathway Analysis (IPA) software (Ingenuity Systems). All connections made in the networks are based on previously published results.

### Western blotting

Cells harvested at different time points were lysed and total protein extracts were used for western blotting using antibodies specific for AR (US Biological, Salem, MA), PSA (Santa Cruz Biotechnology, Dallas, TX), Cbl (C-15) (Santa Cruz Biotechnology, Dallas, TX), TRAF6 (Millipore, Temecula, CA), p27Kip1 (C-19) (Santa Cruz, Biotechnology), IRAK1 (F-4) (Santa Cruz, Biotechnology), ZFAND1 (A-14) (Santa Cruz Biotechnology), FGD4 (Epitomics, Burlingame, CA), ABHD3 (Biorbyt, Cambrige, UK), DOK4 (C-16) (Santa Cruz Biotechnology), EGFR (1005) (Santa Cruz Biotechnology), VEGFA (A-20) (Santa Cruz Biotechnology), α-tubulin (Cell Signaling, Danvers, MA), GAPDH (Sigma-Aldrich, St. Louis, MO). Total extracts (30–50 μg) were directly mixed with Lammeli sample buffer and separated on SDS-PAGE. Immunoblotting was performed using appropriate primary and horseradish peroxidase conjugated respective secondary antibodies. Positive signals were detected using a chemiluminiscence ECL kit (Pierce, Rockford, IL).

## Abbreviations

ADT: Androgen deprivation therapy; AR: Androgen receptor; MiRNA: MIcroRNA; AD: androgen dependent; AI: Androgen independent; CRPC: castration resistant prostate cancer; AS: Androgen sensitive; -104S: LNCaP-104S cells; -104R1: LNCaP-104R1 cells; CDX: Casodex; CDXR: Casodex resistant; V plot: Volcano plot; CSFBS: Charcoal stripped FBS; FC: Fold change; PSA: Prostate specific antigen.

## Competing interests

The author(s) declare that they have no competing interests.

## Authors’ contributions

RO performed cell treatments, profiling and all validation experiments including western blots. He also did the hierarchical clustering and in silico analysis of the targets. He participated in manuscript writing. CN analyzed data using IPA software and performed cell treatment related experiments. RL analyzed data including data normalization, z score calculation and Venn diagrams. RC conceived the idea, wrote the manuscript and performed data analysis. All authors read and approved the final manuscript.

## Supplementary Material

Additional file 1: Figure S1 Light micrograph images of LNCaP cells before and during treatment with CS-FBS and CDX. Click here for file

Additional file 2: Table S1 Listing normalized and log-transformed values of the miRNA expression. Click here for file

Additional file 3: Figure S2 Hierarchical clustering of the data from genome wide miRNA profiling. Click here for file

Additional file 4: Table S2 Listing the p-values, expression patterns and IDs of significant miRNAs identified in t-tests (supple Figure 2). Click here for file

Additional file 5: Figure S3 Volcano plots of the two samples t-tests of the normalized values of untreated and treated LNCaP cells. Click here for file

Additional file 6: Table S3 Listing the log-transformed fold change values of the up regulated and down regulated miRNAs identified in miRNA profiling. Click here for file

Additional file 7: Table S4 Listing miRNAs in specific clusters identified in K-median cluster analysis. Click here for file

Additional file 8: Table S5 Listing the p-values, expression profile and IDs of significant miRNAs from the list of validated miRNAs identified in two samples t-tests. Click here for file

Additional file 9: Table S6 Listing the log-transformed values of the fold change in expression of the validated miRNAs. Click here for file

Additional file 10: Table S7 Listing the up and down regulated subset of the validated miRNAs in specific clusters identified in K-median cluster analysis. Click here for file

Additional file 11: Figure S4 Analysis of association of deregulated miRNAs with canonical pathways and cellular processes. Click here for file

Additional file 12 Supplemental methods and figure legends. Click here for file
