# Supplementary figures and images for: MicroRNA expressions associated with progression of prostate cancer cells to antiandrogen therapy resistance
Source: Mol Cancer. 2014 Jan 3;13:1. doi: 10.1186/1476-4598-13-1 (PMC3896800; doi:10.1186/1476-4598-13-1)

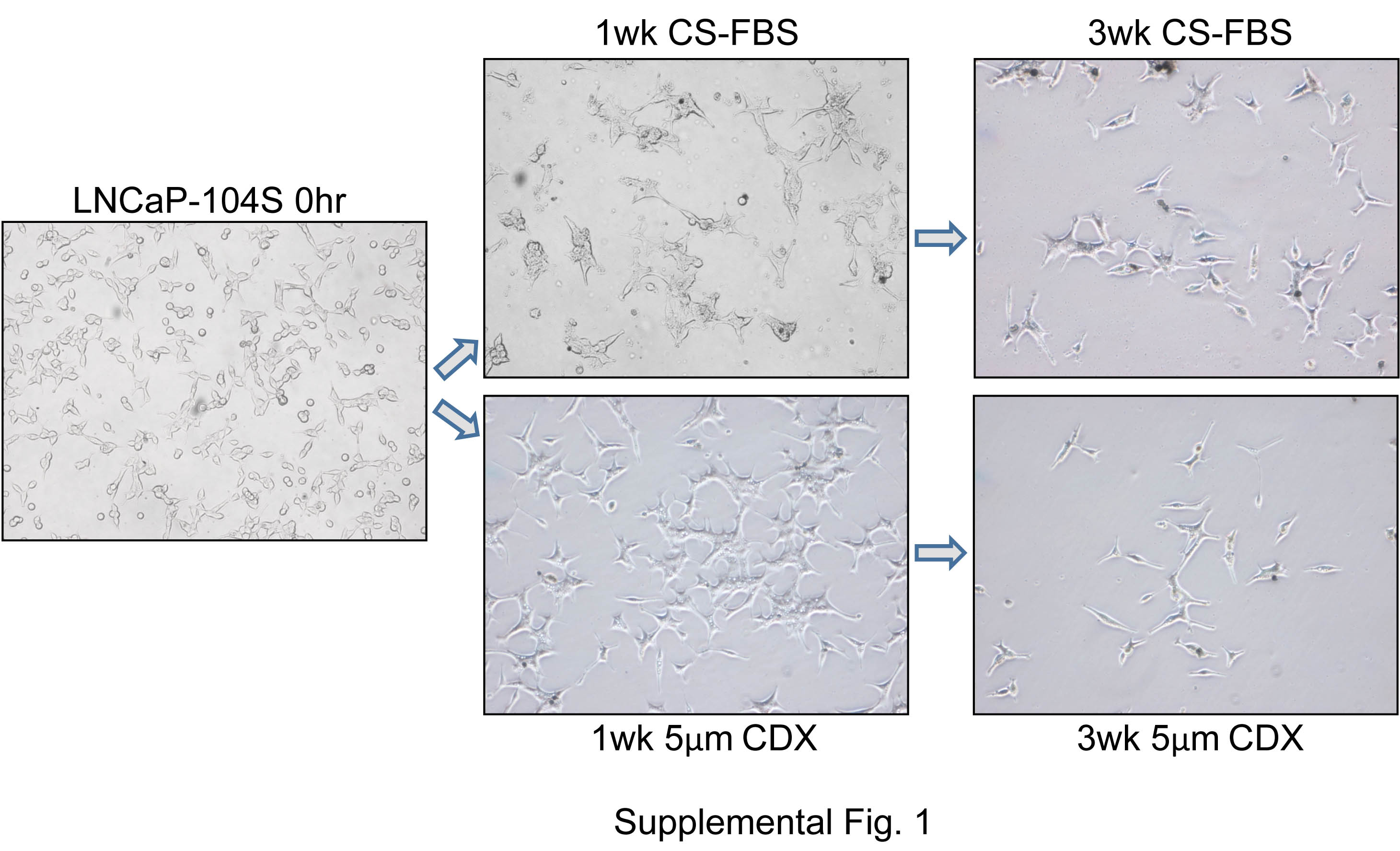

Supplement: Additional file 1: Figure S1 — Light micrograph images of LNCaP cells before and during treatment with CS-FBS and CDX. [file 1476-4598-13-1-S1.jpeg]

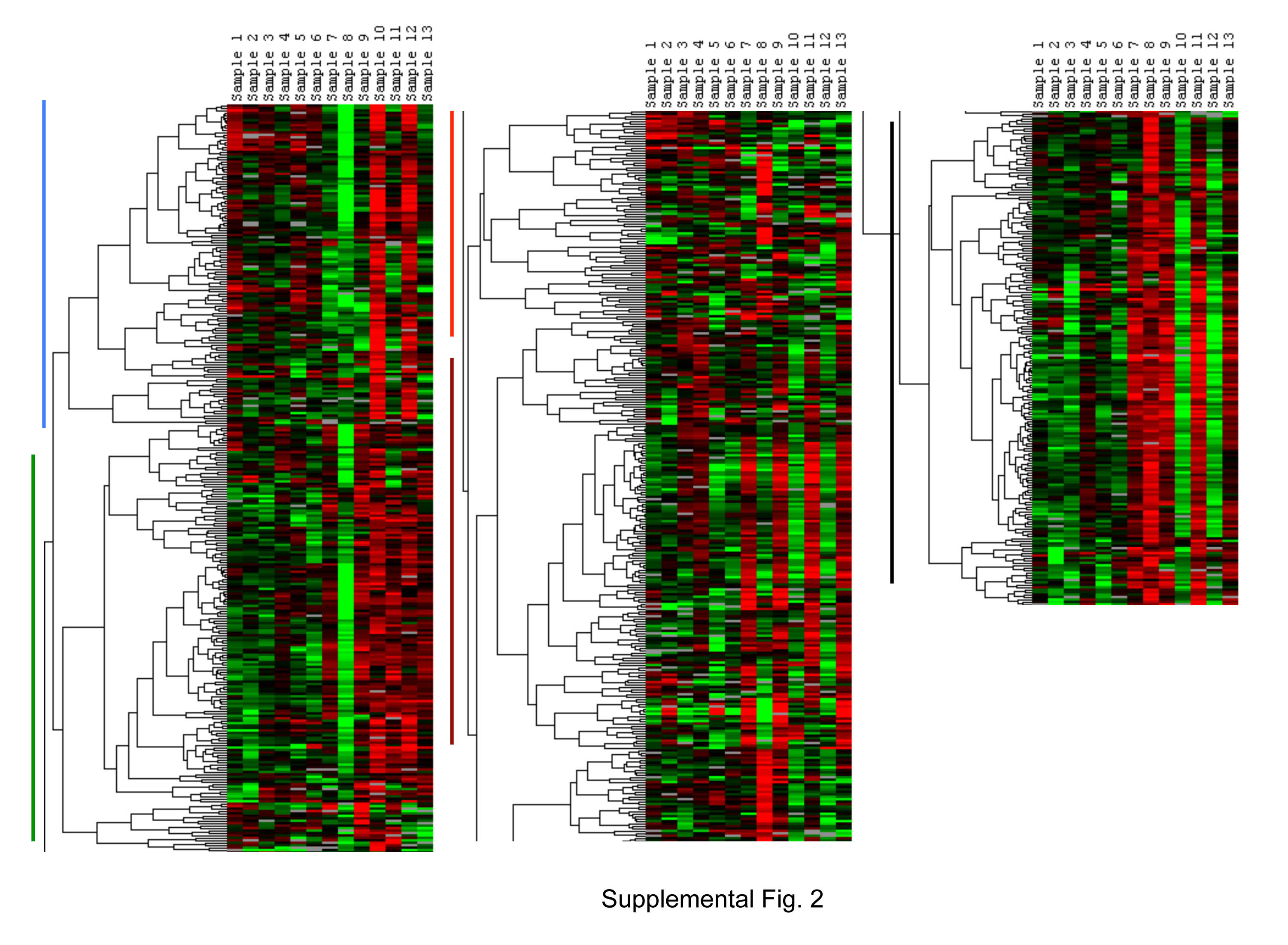

Supplement: Additional file 3: Figure S2 — Hierarchical clustering of the data from genome wide miRNA profiling. [file 1476-4598-13-1-S3.jpeg]

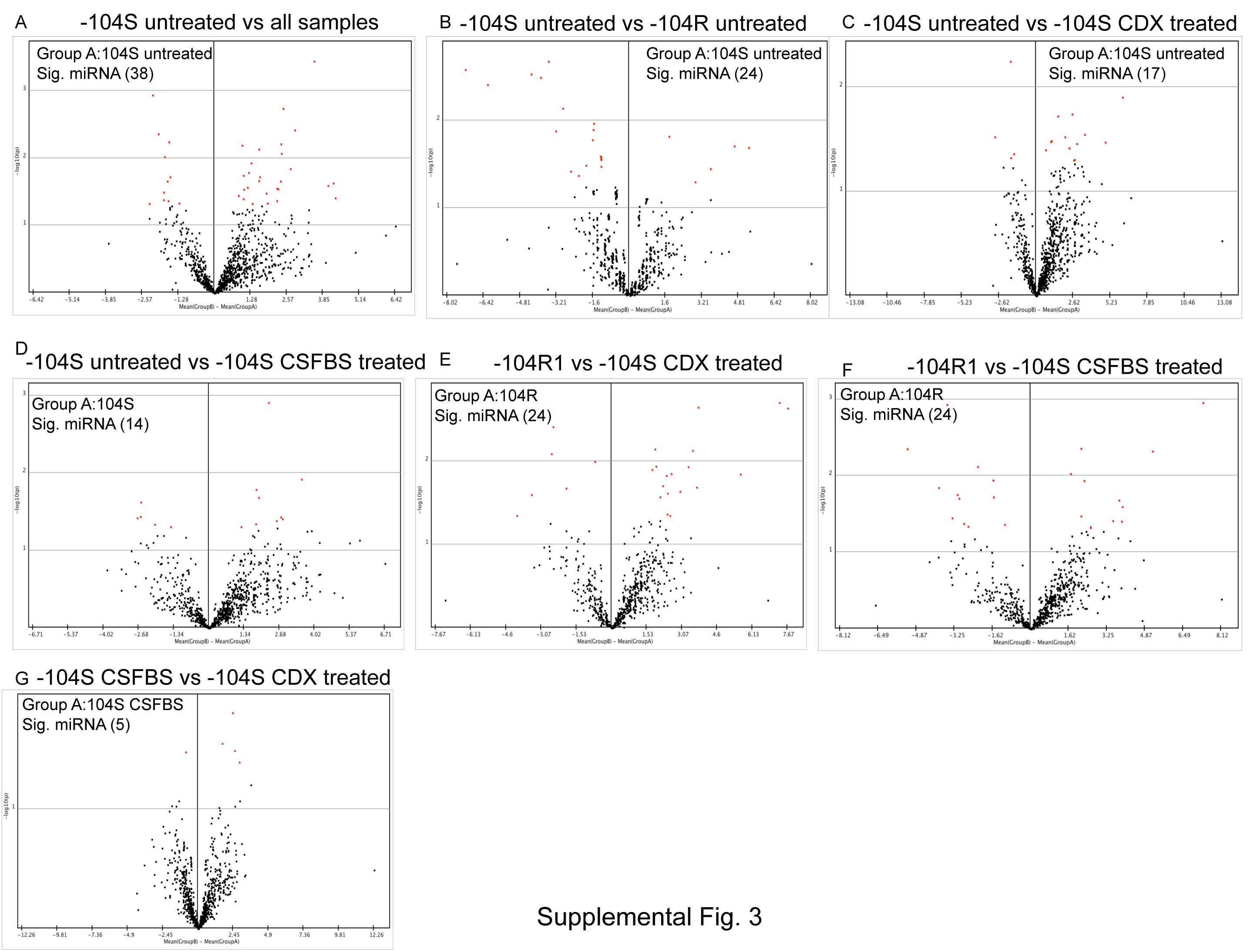

Supplement: Additional file 5: Figure S3 — Volcano plots of the two samples t-tests of the normalized values of untreated and treated LNCaP cells. [file 1476-4598-13-1-S5.jpeg]

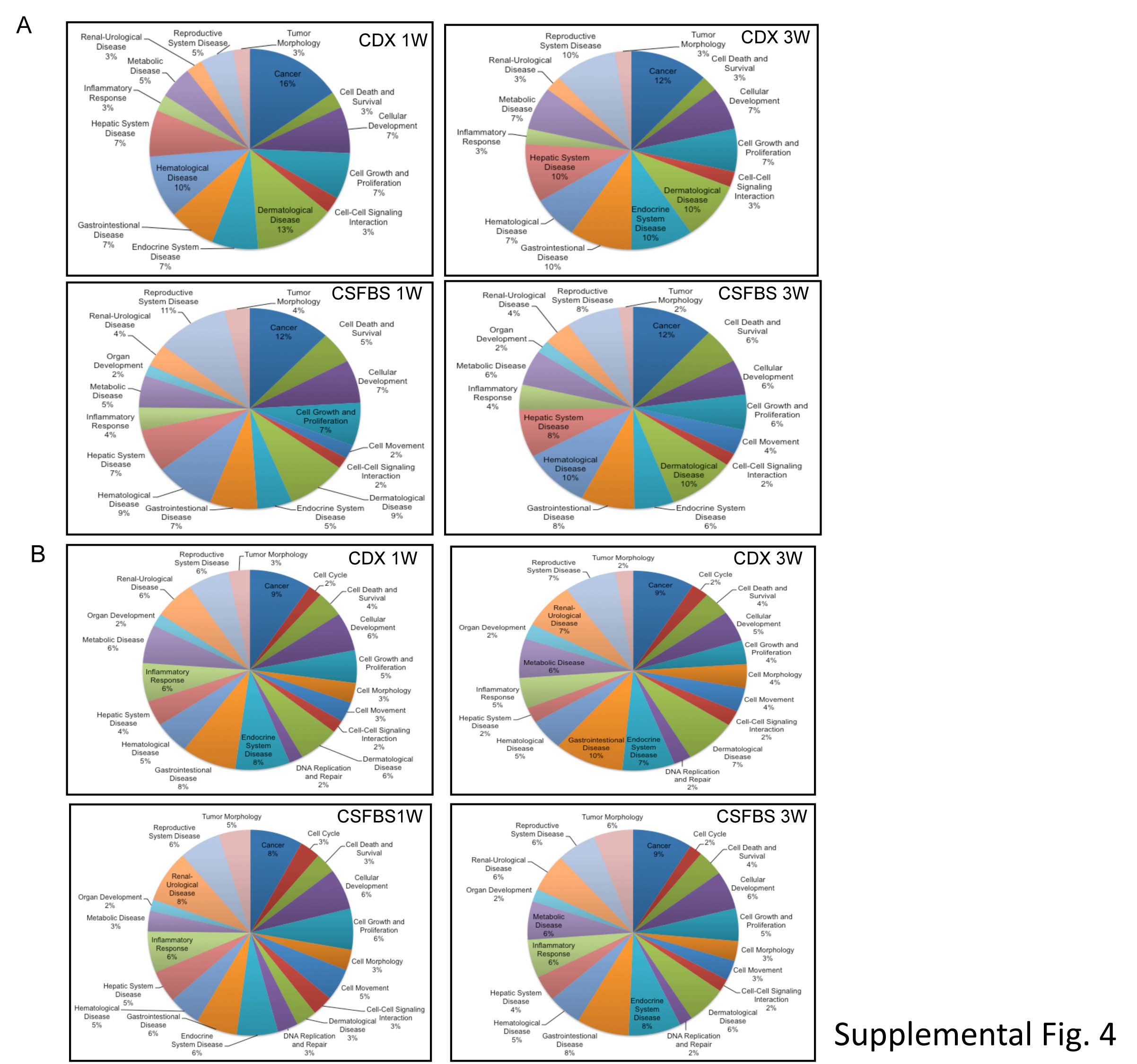

Supplement: Additional file 11: Figure S4 — Analysis of association of deregulated miRNAs with canonical pathways and cellular processes. [file 1476-4598-13-1-S11.jpeg]
