# Supplementary material for: MicroRNA expressions associated with progression of prostate cancer cells to antiandrogen therapy resistance
Source: Mol Cancer. 2014 Jan 3;13:1. doi: 10.1186/1476-4598-13-1 (PMC3896800; doi:10.1186/1476-4598-13-1)
Supplement: Additional file 12 — Supplemental methods and figure legends. [file 1476-4598-13-1-S12.pdf]

Supplemental Methods:

LNCaP Treatment Protocol

To start treatment, $1x10^6$ LNCaP-104S cells were seeded into 10cm dishes containing CS-FBS media. After 24 hrs media was changed to fresh CS-FBS media containing 10% spent media with or without 5um CDX. Treated cells were subcultured initially in every 2 days. By the end of the first week, cell growth has been retarded and during the second week many cells died. An aliquot of treated cells were harvested after one week of treatment. Remaining cells were reseeded at $1x10^6$ cells per dish every 4 days and fresh media was added in between subculturing. During the third week of treatment the cells treated with CS-FBS media started to recover and grew in a more uniform fashion, compared to CDX treated cell population where growth was limited to clonal clusters. At the end of third week, all viable cells were harvested and used for RNA and protein extraction.

Normalization of qRT-PCR Expression Values

During profiling of 1,113 miRNAs by qRT-PCR, each plate contained the same 3 control primers (U6 snRNA, RNU43 snoRNA, RNU1A snRNA) provided with the kit. SDS 2.3 software from ABI generates Ct values for each primer. Ct values for all miRNAs in all samples were normalized to the three internal control primers using qBasePlus software (Biogazelle). Using the Genorm software paired with with qBasePlus, 7 additional stably expressed miRNAs were identified. The Ct values for all miRNAs were then renormalized to mean of the 10 reference targets (3 control primers + 7 identified miRNAs). Following normalization, all the reference primers were again evaluated to ensure their relative miRNA expression values didn’t change. For

validation of expression values, qRT-PCR was conducted in triplicate with a larger reaction volume to minimize experimental error. During validation all ten reference-primers were included in each reaction plate [1].

Fold Change Calculation

Fold change values were calculated to determine the relative changes in miRNA expression as treatment progressed and also to compare relative changes between treatments. These values were generated using the miRNome analysis software from SBI. The values were calculated by first determining the geometric mean, of the ten reference primers, for both samples in the comparison. The geometric means were compared using the formula

ΔCt control= $2^{-(GM\mathbf{c}-GM\mathbf{r})}$

Where GM**c** is the geo-mean of the control sample and GM**r** is the geo-mean of the reference sample. The fold change, or $\Delta\Delta$Ct, for each miRNA was then calculated using the formula

$\Delta\Delta$Ct= $2^{-(Ct\mathbf{R}-Ct\mathbf{C})}$*($\Delta$Ct control)

Where Ct**R**= Reference sample miRNA Ct value, and Ct**C**=Control sample miRNA Ct value.

Z-score Calculation

Z-scores were calculated to rank miRNAs for use in selecting candidates for further investigation. This was accomplished by, first determining the $\Delta\Delta$Ct ($\mathbf{A}_i$) of all miRNAs and using these values to derive the Global mean (**g**).

$$g = \sqrt[n]{\prod_{i=1}^{n} X_i}$$

The global mean is then used to determine the global standard deviation for each comparison using the formula

$$\sigma_g = e^{\sqrt{\frac{\sum_{i=1}^{n}(\ln A_i - \ln g)^2}{n}}}$$

From which z-scores were determined using the formula.

$$z = \frac{A - g}{\sigma}$$

Additional Analysis

Hierarchal clustering was conducted using Cluster 3.0 [2,3] and heat maps were generated by Java TreeView software. Multiexperiment Viewer software (MEV) was used for K-means clustering [4], T-tests [5], and volcano plots.

Supplemental Figure Legend:

Supplemental figure 1: Light micrograph images of LNCaP cells before and during treatment with CS-FBS and CDX.

Supplemental figure 2: Hierarchical clustering of the data from genome wide profiling of miRNAs in LNCaP Cells with or with treatment with CS-FBS and CDX. Samples 1, 2: LNCaP-104R1, Samples 3-5: LNCaP-104S1, Samples 6, 7: LNCaP-104S 1 wk CDX treated, Samples 8, 9: LNCaP-104S 1 wk CSFBS treated, Samples 10, 11: LNCaP-104S 3 wks CDX treated, Samples 112, 13: LNCaP-104S 3 wks CSFBS treated.

Supplemental figure 3: Volcano plots of the two samples t-tests of the normalized values of untreated and treated LNCaP cells as shown in supplemental data 2.

Supplemental figure 4: Analysis of association of deregulated miRNAs with canonical pathways and cellular processes. A) Pie charts showing the percentage of validated up regulated miRNAs in various cellular processes based on the published reports. Upper panel depicts the percentage of miRNAs involved in cellular processes and its changes during 1wk and 3wks treatments with CDX. Lower panel shows changes in the percentage of miRNAs involved in cellar processes during 1wk and 3wks treatments with CSFBS. B) Pie charts showing the percentage of validated down regulated miRNAs in various cellular processes. Upper panel illustrates the changes in the percentage of miRNAs involved in cellular processes during 1wk and 3wks treatment with CDX. Lower panel represents changes in the percentage of miRNAs involved in cellular processes during 1wk and 3wks treatments with CSFBS.

References

1. B D'Haene, P Mestdagh, J Hellemans, J Vandesompele. miRNA expression profiling - from reference genes to global mean normalization. 2011, Methods in Molecular Biology 18:261-272

2. De Hoon, M. J. L., Imoto, S., Nolan, J., and Miyano, S. (2004). Open source clustering software. Bioinformatics, 20 (9), 1453–1454.

3. Eisen, M. B., Spellman, P. T., Brown, P. O., and Botstein, D. (1998). Cluster analysis and display of genome-wide expression patterns. *Proc Natl Acad Sci USA* 95, 14863–14868.

4. Soukas, A., P. Cohen, N.D. Socci, and J.M. Friedman. 2000. Leptin-specific patterns of gene expression in white adipose tissue. *Genes Dev. 14:963-980.*

5. Pan, W. (2002). A comparative review of statistical methods for discovering differentially expressed genes in replicated microarray experiments. *Bioinformatics 18: 546-554.*
